# The Impact of Vaccination and Prior Exposure on Stool Shedding of *Salmonella* Typhi and *Salmonella* Paratyphi in 6 Controlled Human Infection Studies

**DOI:** 10.1093/cid/ciy670

**Published:** 2018-09-25

**Authors:** Malick M Gibani, Merryn Voysey, Celina Jin, Claire Jones, Helena Thomaides-Brears, Elizabeth Jones, Philip Baker, Marcus Morgan, Alison Simmons, Melita A Gordon, Vincenzo Cerundolo, Virginia E Pitzer, Brian Angus, Myron M Levine, Thomas C Darton, Andrew J Pollard

**Affiliations:** 1 Oxford Vaccine Group, Department of Paediatrics, University of Oxford; 2 Nuffield Department of Primary Care Health Sciences, University of Oxford, United Kingdom; 3 Oxford University Hospitals, National Health Service Foundation Trust, United Kingdom; 4 Medical Research Council Human Immunology Unit, Radcliffe Department of Medicine, University of Oxford, United Kingdom; 5 Translational Gastroenterology Unit, University of Oxford, United Kingdom; 6 Malawi-Liverpool-Wellcome Trust Clinical Research Programme, Blantyre; 7 Institute for Infection and Global Health, University of Liverpool, United Kingdom; 8 Department of Epidemiology of Microbial Diseases, Yale School of Public Health, New Haven, Connecticut; 9 Nuffield Department of Medicine, University of Oxford, United Kingdom; 10 Center for Vaccine Development, University of Maryland School of Medicine, Baltimore; 11 Department of Infection, Immunity and Cardiovascular Disease, University of Sheffield; 12 National Institute for Health Research Oxford Biomedical Research Centre, United Kingdom

**Keywords:** stool shedding, Salmonella Typhi, indirect effects, typhoid conjugate vaccine, Vi-polysaccharide vaccine

## Abstract

**Background:**

Shedding of *Salmonella* Typhi or Paratyphi in the stool or urine leads to contamination of food or water, which is a prerequisite for transmission of enteric fever. Currently, there are limited data on the effect of vaccination or prior exposure on stool shedding.

**Methods:**

Six *Salmonella* Typhi or Paratyphi human challenge studies were conducted between 2011 and 2017. Participants were either unvaccinated or vaccinated with 1 of 4 vaccines: Vi-polysaccharide (Vi-PS), Vi-tetanus-toxoid conjugate vaccine (Vi-TT), live oral Ty21a vaccine, or an experimental vaccine (M01ZH09). Daily stool cultures were collected for 14 days after challenge.

**Results:**

There were 4934 stool samples collected from 430 volunteers. Participants who received Vi-PS or Vi-TT shed less than unvaccinated participants (odds ratio [OR], 0.34; 95% confidence interval [CI], 0.15–0.77; *P* = .010 and OR, 0.41; 95% CI, 0.19–0.91, *P* = .029 for Vi-PS and Vi-TT, respectively). Higher anti-Vi immunoglobulin G titers were associated with less shedding of *S*. Typhi (*P* < .0001). A nonsignificant reduction in shedding was associated with Ty21a vaccine (OR, 0.57; 95% CI, 0.27–1.20; *P* = .140). Individuals previously exposed to *S*. Typhi shed less than previously unexposed individuals (OR, 0.30; 95% CI, 0.1–0.8; *P* = .016). Shedding of *S*. Typhi was more common than *S*. Paratyphi.

**Conclusions:**

Prior vaccination with Vi vaccines, or natural infection, reduces onward transmission of *S*. Typhi. Field trials of Vi-TT should be designed to detect indirect protection, reflecting the consequence of reduced stool shedding observed in the human challenge model.

## Teaser

Six *Salmonella* Typhi or Paratyphi human challenge studies were conducted, and daily stool cultures performed. Vi-containing vaccines reduced bacterial shedding, Ty21a or an experimental vaccine did not. Higher Vi immunoglobulin G titers were associated with reduced shedding.

Infection with *Salmonella enterica* subspecies *enterica* serovars Typhi or Paratyphi (*S.* Typhi or *S.* Paratyphi) is estimated to be responsible for between 11.6 and 26.9 million cases of enteric fever and 75000–216510 deaths annually [1–5]. Transmission of *S.* Typhi and *S.* Paratyphi occurs primarily through consumption of contaminated food or water, via short-cycle or long- cycle transmission. Short-cycle transmission is defined as the contamination of food and water in the immediate environment, whereas long-cycle transmission is defined as contamination of the broader environment, such as pollution of water supplies by sewage or inadequate treatment of piped water [6]. The relative contribution of each transmission mode may vary depending on the epidemiological context and may differ between *S*. Typhi and *S.* Paratyphi [7]. As these serovars are human restricted, all modes of transmission involve shedding of the organism by infected individuals during incubation, acute disease, or convalescence or by chronic long-term carriers, ultimately resulting in contamination of food or water consumed by susceptible individuals. Disease control is therefore likely to require the integration of initiatives to improve water quality, sanitation, and hygiene, coupled with the deployment of effective vaccines [8].

Vaccines that both protect against clinical disease as well as reduce shedding would likely have enhanced effectiveness by interrupting transmission and providing indirect protection to unvaccinated individuals. Live attenuated oral typhoid vaccine, Ty21a, reduced stool shedding in the early Maryland challenge studies [9] and appears to induce herd immunity in field trials [10]. However, there are conflicting data on the indirect protection conferred by Vi-polysaccharide vaccines (Vi-PS) [11, 12] and limited data on the impact of new Vi-tetanus-toxoid conjugate vaccines (Vi-TT) on stool shedding [13]. Finally, the impact of previous exposure to typhoidal *Salmonella* on stool shedding after subsequent exposure has not been previously explored.

Experimental challenge studies of closely monitored volunteers can be used to describe microbial dynamics in clinical and subclinical typhoid and paratyphoid infections, including the timing and pattern of stool shedding after challenge in naive or vaccinated individuals [14]. Early experimental human challenge studies in Maryland indicate that shedding is more common in individuals who develop typhoid disease. However, individuals who fail to develop disease following challenge can continue to shed bacteria for several weeks [15]. Differences in stool shedding patterns between *S*. Typhi and *S.* Paratyphi A are poorly understood. Improved estimates of stool shedding dynamics in enteric fever are needed, particularly in the context of different immune states, as these form important variables in models of typhoid transmission dynamics and estimates of vaccine impact [16, 17].

We performed an analysis of stool shedding dynamics in healthy volunteers enrolled into closely monitored *S*. Typhi and *S.* Paratyphi human challenge studies. Our aims in this study were to model stool shedding after experimental challenge and to compare those challenged with *S*. Typhi vs *S*. Paratyphi, participants who did or did not develop enteric fever after challenge, those who received typhoid vaccines vs those who were unvaccinated, rechallenged individuals previously exposed to *S*. Typhi or *S*. Paratyphi vs previously unexposed individuals, differences according to demographic variables, and the relationship between antibody levels and stool shedding.

## METHODS

### Typhoid and Paratyphoid Human Challenge Studies

Data were available from 6 enteric fever human challenge studies conducted in Oxford between 2011 and 2017. A list of included studies is provided in Table 1.

**Table 1.** List of Included Studies

	Study	Challenge Agent	Study Type	Vaccine	Description	References
1	OVG2009/10 (T1)	*Salmonella* Typhi Quailes strain	Observational	…	Dose-finding study Low dose: 1–5 × 10^3^ CFU (n = 20) High dose: 1–5 × 10^4^ CFU (n = 20)	[18]
2	OVG2011/02 NCT01405521 (T2)	*S*. Typhi Quailes strain	Vaccine RCT	M01ZH09 (n = 31)^a^ Ty21a (n = 30)^a^ Placebo (n = 30)	Ty21a (3 dose) or M01ZH09 (single dose) vaccines compared with control Challenge dose 1–5 × 10^4^ CFU 28 days after vaccination	[19]
3	OVG2013/07 NCT02100397 (P1)	*S*. Paratyphi A NVGH308 strain	Observational	…	Dose-finding study High dose: 1–5 × 10^3^ CFU (n = 20) Low dose: 0.5–1 × 10^3^ CFU (n = 20)	[14]
4	OVG2014/08 NCT02324751 (VAST)	*S*. Typhi Quailes strain	Vaccine RCT	Vi-PS (n = 35)^a^ Vi-TT conjugate (n = 37)^a^ Placebo (n = 31)	Single dose Vi-PS (Typhim Vi^®^, Sanofi Pasteur) or Vi-TT (TypbarTCV^®^, Bharat Biotech) vaccines compared with control MenACWY Challenge dose 1–5 × 10^4^ CFU 28 days after vaccination	[13]
5	OVG2014/01 NCT02192008 (PATCH)	*S*. Paratyphi A NVGH308 strain *S*. Typhi Quailes strain	RCT	…	Naive challenge (*S*. Typhi and *S*. Paratyphi) vs rechallenge (homotypic and heterotypic) *S.* Typhi challenge dose 1–5 × 10^4^ CFU *S.* Paratyphi challenge dose 1–5 × 10^3^ CFU	[20]
6	OVG2016/03 NCT03067961 (TYGER)	*S*. Typhi Quailes strain and *S*. Typhi SB6000 (TT deficient)	RCT	…	Wild-type *S*. Typhi Quailes strain (n = 20) SB6000 TT negative strain (n = 20) Challenge dose 1–5 × 10^4^ CFU	[21]

Abbreviations: CFU, colony-forming unit; PS, polysaccharide; RCT, randomized controlled trial: TT, tetanus-toxoid.

^a^Vaccinated and completed challenge.

All challenge studies followed comparable protocols, detailed elsewhere (Supplementary Materials) [13, 18, 19, 20, 21, 22]. Briefly, healthy adults drank 120 mL of sodium bicarbonate solution prior to challenge. After challenge, daily blood and stool cultures were collected for 14 days. Participants were diagnosed with enteric fever if they had fever of 38^o^C for ≥12 hours and/or *S*. (Para)Typhi bacteremia detected ≥72 hours from challenge. Antibiotics were initiated at the time of diagnosis or at day 14 for those not diagnosed. All participants were effectively treated, and no chronic carriers were identified.

Typhoid challenge was performed using the Qualies strain (genotype 3.1.0) [15, 23]. Paratyphoid challenge was performed using the *S.* Paratyphi A NVGH308 strain [14]

### Stool Culture

Stool cultures were performed according to local procedures based on national guidance at Oxford University Hospital National Health Service (NHS) Foundation Trust (Supplementary Materials) [24].

### Antibody Measures

Anti-Vi immunoglobulin (Ig) G titers were measured using a commercial enzyme-linked immunosorbent assay (ELISA) kit (VaccZyme, The Binding Site, Birmingham, UK) according to the manufacturer’s guidelines [13]. IgG and IgA isotype responses to *S*. Typhi lipopolysaccharide (LPS) (Sigma L2387), *S*. Typhi H*d* (University of Maryland 01-CVD0150622-01), *S*. Paratyphi A O:2 (GSK Vaccines for Global Health) [25], and *S*. Paratyphi H*a* (University of Maryland CVD 1902D lot CVD141113-01) antigens were measured with an in-house ELISA in serum samples collected immediately prior to challenge [14, 18, 19].

### Statistical Analyses

Stool culture data were combined in mixed effects logistic regression models, which included participant-specific random intercepts to account for the multiple samples per person. Models were adjusted for the vaccine received, study, and, where applicable, challenge dose (high or low). “Day” was included in the model as a categorical factor to allow the odds of stool shedding to vary by day.

An overall interaction term (day-by-vaccine) was tested to determine if vaccination altered the pattern of shedding over time. An interaction term for day-by-diagnosis status was used to compare the pattern of shedding in those who were diagnosed with enteric fever and those who remained undiagnosed at day 14.

The linear predictor from the model was exported for each participant for each day and converted into a probability by taking the inverse logit. These probabilities are presented in figures with a loess smooth to illustrate the findings from the logistic regression models. Odds ratios (ORs) presented from logistic regression models represent the ratio of the odds of shedding in comparative groups on average across all 14 days.

All models were fitted in SAS version 9.4, and code is displayed in the Supplementary Materials.

## RESULTS

In total, 4934 stool samples from 430 participants were analyzed; 3698 samples were from *S*. Typhi challenge participants and 1236 samples were from *S*. Paratyphi challenge participants (Table 2). In total, 14.5% of stool samples from *S*. Typhi-challenged participants were positive compared with 7.5% of samples from those challenged with *S*. Paratyphi A.

**Table 2.** Stool Microbiology by Challenge Agent and Study

	Study	Number of Participants	Number of Participants With ≥1 Positive Stool Sample	Number of Days Shedding (Median [Interquartile Range])	Number of Samples from *Salmonella* Typhi-challenged Participants		Number of Samples from *Salmonella* Paratyphi A-challenged Participants		Total
					Negative	Positive	Negative	Positive	
1	OVG2009/10 (T1)	40	22 (55%)	1 [0–2]	520	54 (9%)	...	...	574
2	OVG2011/02 (T2)	92	72 (78%)	2 [1–4]	908	217 (19%)	...	...	1125
3	OVG2013/07 (P1)	40	17 (43%)	0 [0–1]	...	...	510	36 (7%)	546
4	OVG2014/08 (VAST)	103	64 (62%)	1 [0–2]	842	126 (13%)	...	...	968
5	OVG2014/01 (PATCH)	115	56 (45%)	0 [0–2]	562	84 (13%)	633	57 (8%)	1336
6	OVG2016/03 (TYGER)	40	24 (60%)	1 [0–2]	329	56 (15%)	...	…	385
	**Total**	**430**	**255 (59%**)		**3161**	**537 (14.5%**)	**1143**	**93 (7.5%**)	**4934**

### Differences in *S.* Typhi Stool Shedding According to Diagnosis Status

**Figure 1.**
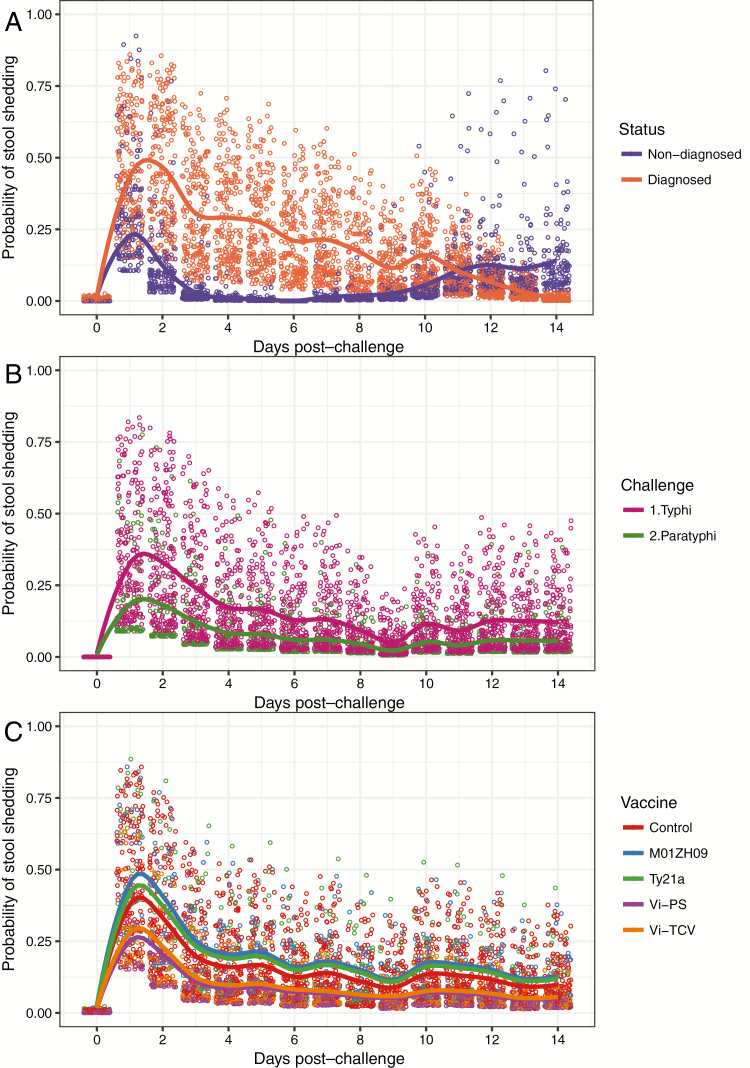
Probability of bacterial shedding in stool by day in controlled human infection enteric fever studies. *(A*) N = 331 participants challenged with *Salmonella* Typhi according to diagnosis status. Nondiagnosed N = 145, diagnosed N = 186. *(B*) Unvaccinated participants exposed to oral challenge with *S*. Typhi (N = 197) or *S*. Paratyphi (N = 109) bacteria. *(C*) Vaccinated and unvaccinated participants challenged with 1–5 × 10^4^ colony-forming units *S*. Typhi wild-type bacteria according to vaccine received. Control vaccine or no vaccine (N = 158); M01ZH09, experimental typhoid vaccine (N = 32); Ty21a, live attenuated oral typhoid vaccine (N = 30); Vi-PS, Vi-polysaccharide typhoid vaccine (Typhim Vi^®^, Sanofi Pasteur; N = 35); Vi-TT, Vi-tetanus toxoid conjugate vaccine (TypbarTCV^®^, Bharat Biotech; N = 37)

There were 331 participants challenged with *S*. Typhi, of whom 186 (56.2%) met the diagnostic criteria for typhoid fever [18]. The highest incidence of shedding was observed on day 1 and day 2 after challenge. The pattern of shedding over time was significantly different between those diagnosed and those who did not develop enteric fever (*P* < .0001 day-by-diagnosis interaction). The odds of shedding 1 day after challenge were 2.5 times greater in those who were later diagnosed than in those who remained undiagnosed (OR, 2.49; 95% confidence interval [CI], 1.32–4.69; *P* = .0049). On day 2 odds were 9 times higher in those later diagnosed (OR, 8.93; 95% CI, 3.86–20.61; *P* < .0001) and on day 3 were 23 times higher (OR, 22.59; 95% CI, 6.83–74.7; *P* < .0001). In the second week after challenge, many diagnosed participants had commenced antibiotics, and shedding ceased. In the undiagnosed participants, increased shedding was observed from day 10 onward, until those participants also received treatment on day 14 (Figure 1A). A similar pattern was observed in historic challenge studies (**Supplementary Figure S1**).

### Differences in Stool Shedding After *S*. Typhi or *S*. Paratyphi Challenge in Unvaccinated Participants

There were 197 unvaccinated participants exposed to *S*. Typhi and 109 unvaccinated participants exposed to *S*. Paratyphi. The odds of shedding in participants exposed to *S*. Typhi were twice as high as in those exposed to *S*. Paratyphi (OR, 1.97; 95% CI, 1.00–3.88; *P* = .049). A sensitivity analysis excluding low-dose challenge gave a very similar estimate (OR, 1.97; *P* = .044). The dose received was nonsignificant in the model (Figure 1B).

### Effect of Vaccination on Stool Shedding After *S*. Typhi Challenge

Data from 5 *S*. Typhi studies were analyzed. Participants challenged with a low dose or a genetically modified *S*. Typhi were excluded [21].

Participants who received Vi-PS or Vi-TT vaccine had lower rates of shedding than unvaccinated participants (OR, 0.34; 95% CI, 0.15–0.77; *P* = .010 and OR, 0.41; 95% CI, 0.19–0.91; *P* = .029 for Vi-PS and Vi-TT, respectively). In Ty21a vaccine recipients, there was a nonsignificant reduction in shedding compared with unvaccinated controls (OR, 0.57; 95% CI, 0.27–1.20; *P* = .14; Figure 1C). There were no differences between unvaccinated participants and those who received the M01ZH09 vaccine or between any other groups after adjusting for study-specific variation. The vaccine-by-day interaction term was nonsignificant, showing that the pattern of shedding over time was similar for all vaccines even though the amount of shedding differed.

### Effect of Previous Exposures

**Figure 2.**
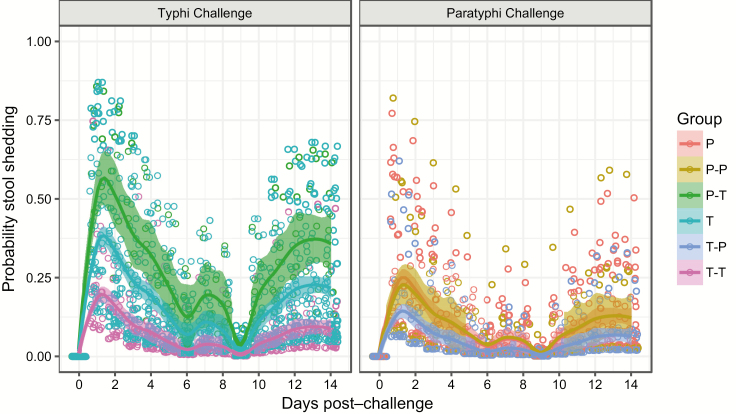
Bacterial shedding in stool after *S*. Typhi or *S*. Paratyphi challenge, according to previous exposure. P = *S*. Paratyphi naive (n = 39); P-P = *S*. Paratyphi rechallenge after previous *S.* Paratyphi exposure (n = 13); P-T = *S*. Typhi challenge after previous *S*. Paratyphi exposure (n = 10); T = *S*. Typhi challenge in *S*. Typhi naive participants (n = 71); T-P = *S*. Paratyphi challenge after previous *S*. Typhi exposure (n = 27); T-T = *S*. Typhi rechallenge after previous *S*. Typhi exposure (n = 27). See Supplementary Table S1 for model outputs.

Participants previously exposed to *S*. Typhi had less stool shedding than previously unexposed individuals (T-T vs T, OR, 0.33; 95% CI, 0.1–0.8; *P* = .016; Figure 2). For those participants who received *S*. Typhi challenge, previous exposure to *S*. Paratyphi significantly increased stool shedding compared with previous *S*. Typhi exposure (P-T vs T-T: OR, 7.5; 95% CI, 2.0–28.4; *P* = .003) and nonsignificantly increased the rate of bacterial shedding compared with previously unexposed individuals (P-T vs T: OR, 2.5; 95% CI, 0.8–8.0; *P* = .125). For those receiving *S*. Paratyphi challenge, there were no differences between groups, and a lower rate of shedding in general was observed in comparison with those exposed to *S*. Typhi challenge.

### Variation in Shedding According to Age and Sex

In 197 unvaccinated participants (34% female), the median number of samples provided was 11 (interquartile range [IQR], 8–14) for women and 12 (IQR, 9–15) for men. Men had proportionately more positive stool samples than women (OR, 1.78; 95% CI, 1.10–2.87; *P* = .0187). Age was not significantly related to shedding (*P* = .795).

### Antibody Levels Prior to Challenge

**Figure 3.**
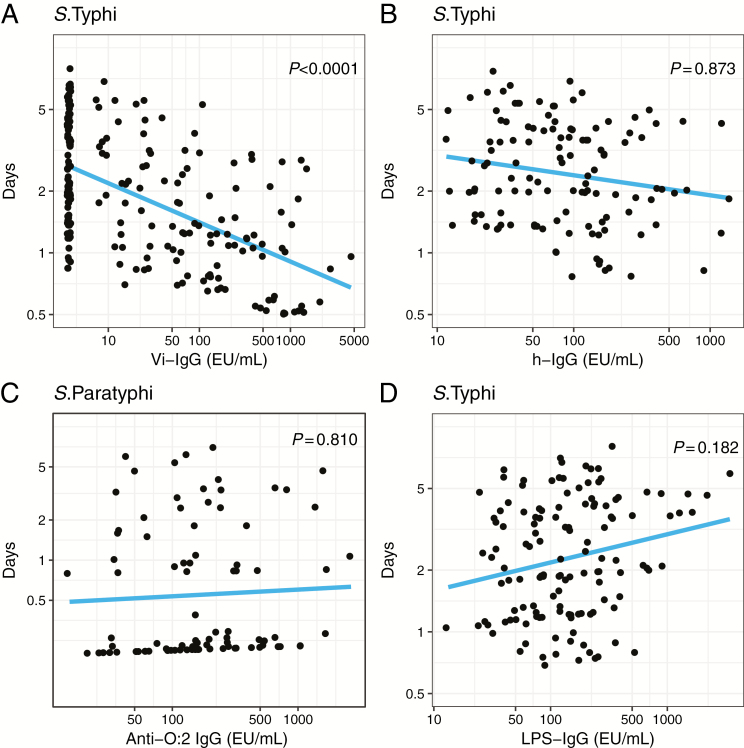
Relationship between days of bacterial shedding in stool after *Salmonella* Typhi or *S*. Paratyphi challenge and antibody levels prior to challenge. *(A*) Anti-Vi immunoglobulin (Ig) G prior to challenge with *S*. Typhi. *(B*) Anti-Hd IgG prior to challenge with *S*. Typhi. *(C*) Anti-O:2 IgG prior to challenge with *S*. Paratyphi. *(D*) Anti-*S*. Typhi lipopolysaccharide IgG prior to challenge with *S*. Typhi. Days: *y*-axis represents the predicted total number of days of stool shedding (out of 14). The total number of days was determined from the logistic regression model by summing across all 14 days the predicted probability for each day for each person. Abbreviation: Ig, immunoglobulin.

Higher anti-Vi IgG antibody titers prior to challenge were associated with less bacterial shedding after challenge with *S*. Typhi (*P* < .0001). There was no relationship between anti-LPS IgG or anti-H*d* IgG and shedding after challenge with either *S*. Typhi or *S.* Paratyphi (Figure 3). IgA and IgM responses to *S*. Typhi LPS and H*d* antigens were nonsignificant (Supplementary Figure S2).

## DISCUSSION

Shedding of *S*. Typhi and *S*. Paratyphi bacilli is a prerequisite for onward transmission in these human-restricted infections. This is the first comprehensive analysis of bacterial shedding from almost 5000 stool samples taken after deliberate challenge of healthy volunteers with *S.* Typhi or *S.* Paratyphi. We demonstrate that stool shedding is more common in individuals who meet the case definition of enteric fever (fever 38^o^C for ≥12 hours and/or *S*. (Para)Typhi bacteremia), but shedding can also occur in the absence of bacteremia or clinical symptoms of disease. Vaccination with Vi-PS or Vi-TT significantly reduced stool shedding of *S.* Typhi following controlled human infection, suggesting that these vaccines are likely to reduce onward transmission of disease. The decreased shedding following vaccination with the live-attenuated Ty21a vaccine was not significant, possibly due to the small sample sizes available for these comparisons and moderate protective efficacy of Ty21a in the challenge model. In earlier challenge studies with Ty21a involving larger numbers of participants who received a freshly harvested formulation of vaccine, shedding of *S*. Typhi was significantly reduced in recipients of the live oral vaccine [9].

The effects of Vi-PS or Vi-TT vaccination on indirect protection and stool shedding are poorly understood. A cluster randomized control trial in Kolkata, India, demonstrated that Vi-PS vaccination can result in indirect protection against typhoid fever in unvaccinated individuals resident in population clusters randomized to Vi-PS vaccine [11]. However, indirect protection of Vi-PS was not observed in another cluster randomized trial conducted in Karachi, Pakistan. One difference is that the trial in Kolkata vaccinated adults as well as children [12].

The reduction in stool shedding observed in individuals vaccinated with a Vi-TT conjugate vaccine is an important finding. To date, there are no completed cluster randomized trials of typhoid conjugate vaccines. However, 1 trial is ongoing in Bangladesh, and individually randomized trials are ongoing in Nepal and Malawi [26]. A previous Vi-conjugate vaccine with *Pseudomonas aeruginosa* exotoxin A as a carrier protein was shown to have a high vaccine efficacy (VE) in Vietnam (VE, 91.1%; 95% CI, 78.6%–96.5%) [27]. Vi-TT vaccine is highly immunogenic in children [28], with demonstrated efficacy of 54.6%–87.1% in a controlled human infection model (depending on the diagnostic criteria used) [13] and vaccine efficacy of 85% estimated from serological data [29]. In October 2017 the World Health Organization recommended the introduction of typhoid conjugate vaccines for children aged >6 months in typhoid-endemic countries [8]. It remains to be determined to what degree the reduction in shedding associated with Vi-TT vaccination will translate to indirect protection of unvaccinated persons in field studies. If the reduction in shedding translates to indirect protection of nonvaccinees in field settings, the overall effectiveness of Vi-TT conjugate vaccines could be significantly higher than is estimated in challenge studies.

Current models that predict the potential impact of Vi-conjugate vaccines account for this potential indirect protection by assuming that transmission of *S.* Typhi is reduced in a manner that is proportional to the vaccine efficacy (ie, by preventing infection and hence shedding among a proportion of vaccinated individuals). The results presented here support such assumptions. The protection afforded by Vi-TT against typhoid infection is similar to its effect against stool shedding. When both are expressed as ORs, the effects of Vi-TT against typhoid diagnosis and shedding have largely overlapping CIs (typhoid diagnosis: OR, 0.16; 95% CI, 0.05–0.46 and shedding: OR, 0.41; 95% CI, 0.19–0.91). However, more analysis is needed to examine the relationships between vaccination, stool shedding, and the development of clinical typhoid and how this may vary between the human challenge model and field settings. Additionally, the mechanism by which anti-Vi antibodies prevent stool shedding requires further investigation.

The nonsignificant reductions in shedding with Ty21a vaccine contrast with data from early challenge studies, where Ty21a was associated with a reduction in any stool shedding of *S*. Typhi from between day 4 and day 30 post-challenge (OR, 0.08; 95% CI, 0.02–0.29) [9]. The differences may result from methodological differences between the Oxford and Maryland challenge studies, including mode of administration, pretreatment with sodium bicarbonate instead of milk, differences in challenge dose (Maryland, 10^5^ organisms; Oxford, 10^4^), and criteria for initiating antibiotics. Of note, Ty21a appeared to provide herd immunity in field studies conducted in Chile [10].

As was observed in early typhoid challenge studies, early shedding increased the likelihood of subsequent development of typhoid fever [15]. Interestingly, we observed an increase in shedding in the undiagnosed group from day 10 onward. It is possible that late shedding represents a harbinger of subsequent bacteremia or fever, such that a proportion of the “undiagnosed” group may have developed enteric fever had the infection not been halted by commencing antibiotics at day 14. Conversely, late, asymptomatic shedding has been described in undiagnosed participants from early challenge studies, where shedding peaked in the second week post-challenge before clearing spontaneously by 6 weeks (in the absence of clinical disease or antibiotic treatment) [15]. These data emphasize that a proportion of individuals exposed to typhoidal *Salmonella* will act as asymptomatic short-term carriers who transiently shed in the absence of overt clinical disease; this is a factor that should be considered when determining the target population for vaccination campaigns.

In individuals previously challenged with *S*. Typhi, we detected a significant reduction in the rate of stool shedding compared with naive controls. A single episode of typhoid exposure is thought to confer moderate protection against subsequent clinical disease (estimated at approximately 23% from historical challenge studies [30]), and modeling studies assume that multiple episodes of typhoid exposure are required to induce functional immunity [31]. These are the first data to suggest that prior typhoid exposure significantly affects the pattern of shedding following rechallenge, albeit from a small sample size (n = 27 rechallenged). Interestingly, we observed that individuals previously exposed to *S*. Paratyphi A had substantially higher rates of shedding when rechallenged with *S*. Typhi. The reasons for the increased risk of typhoid shedding on heterotypic rechallenge are unclear and are the subject of ongoing studies focusing on the role of secretory IgA [32].

When shedding rates were compared between typhoid and paratyphoid challenge studies, rates of shedding following *S*. Typhi challenge were twice as high as those following *S*. Paratyphi A challenge. The higher rate of shedding following typhoid challenge may reflect the higher challenge dose administered in the typhoid model (10^4^ vs 10^3^ colony-forming units), or differences may exist between these serovars in host–pathogen interactions and transmission mechanisms. Several studies in areas coendemic for *S*. Typhi and *S.* Paratyphi A have suggested that transmission dynamics and risk factors may differ between the 2 serovars [7, 33, 34]. For example, *S*. Paratyphi A cases appear to be more spatially dispersed than *S*. Typhi cases in an urban area [33] and may be particularly associated with foodborne transmission [7, 34]. Improved understanding of paratyphoid shedding and transmission dynamics will be an important consideration in the development of vaccines for paratyphoid fever [35].

There are several limitations to assessing stool shedding using data from human challenge studies. The population enrolled comprises adults from a nonendemic country; shedding dynamics may differ in individuals from endemic countries with prior immune priming or in children, who represent the majority of enteric fever cases (but not necessarily the majority of shedders). In this analysis, data were pooled across 6 studies conducted over 6 years. While all samples were processed in the same laboratory using consistent protocols, study-to-study variation may still exist in the sensitivity of stool testing; thus, “study” was adjusted for in all models. Furthermore, challenge was conducted using only a single strain of *S*. Typhi (Quailes) or *S.* Paratyphi A (NVGH308) at a single dose, which may not mirror the shedding dynamics of contemporary circulating strains in Asia and Africa, such as the multidrug-resistant associated H58 (genotype 4.3.1) strain of *S*. Typhi [36]. These limitations primarily reflect safety considerations required for controlled human challenge, and these data should be interpreted alongside emerging data from surveillance studies [37].

In summary, we have performed the first detailed model of shedding dynamics in the context of controlled typhoid and paratyphoid challenge and provide evidence for efficacy of new and existing typhoid vaccines (Vi-PS and Vi-TT) in reducing rates of shedding. These studies illustrate the value of closely monitored experimental human challenge studies in obtaining novel insights into host–pathogen interactions and microbial dynamics, which can directly inform the disease control efforts for priority pathogens.

## Supplementary Data

Supplementary materials are available at *Clinical Infectious Diseases* online. Consisting of data provided by the authors to benefit the reader, the posted materials are not copyedited and are the sole responsibility of the authors, so questions or comments should be addressed to the corresponding author.

Supplementary Material

## Notes

***Author contributions.*** M. M. G., C. J., and T. D. coordinated the clinical studies. M. M. G., C. J., T. D., H. T. B., E. J., P. B., and M. M. collected the data. M. V. conceived the study design and analyzed the data. B. A. provided clinical oversight to all studies. M. M. G. and M. V. wrote the manuscript. A. J. P., M. A. G., A. S., and V. C. designed, oversaw, and obtained funding for study OVG2014/01. A. J. P. supervised the work and obtained funding for the 6 studies. All authors reviewed and edited the manuscript and approved the final version.

***Acknowledgments.*** The authors acknowledge the contribution of all participants who took part in the studies described herein. In addition, the authors acknowledge the following persons and groups: all members of the microbiology laboratory at the Oxford University Hospital NHS Foundation Trust; the University of Maryland School of Medicine for provision of *S*. Typhi Hd and *S*. Paratyphi Hd antigens; Dr Laura Martin (GSK Vaccines for Global Health) for provision of the *S.* Paratyphi NVGH308 challenge strain and O:2 antigen; and Prof William E. Woodward for permission to publish data from his 1975 report on the Maryland challenge studies.

***Disclaimer.*** M. V. and M. M. G. had full access to all study data and had final responsibility for the decision to submit the manuscript. The funders had no involvement in the design and conduct of the studies; collection, management, analysis, and interpretation of the data; preparation, review, or approval of the manuscript; and decision to submit the manuscript for publication. The views expressed in this manuscript are those of the authors and do not necessarily reflect the views of the Joint Committee on Vaccination and Immunisation (JCVI), NHS, NIHR, UK the Department of Health, European Medicines Agency (EMA), or the World Health Organization (WHO).

***Funding.*** M. V. is funded by the National Institute for Health Research (NIHR) Doctoral Research Fellowship (DRF-2015-08-048). Studies OVG2009/10 and OVG2011/12 were supported by a Wellcome Trust Strategic Translational Award (grant 092661) and Emergent BioSolutions (challenge strain manufacture, vaccine/placebo supply, and labeling). Study OVG 2013/07 was supported by the Bill & Melinda Gates Foundation (OPP1084259) and the European Vaccine Initiative. Study 2014/08 was supported by the Bill & Melinda Gates Foundation (OPP1084259) and European Commission FP7 grant Advanced Immunization Technologies. Study OVG 2016/03 was funded by the Bill & Melinda Gates Foundation (OPP1126235). Study 2014/01 was funded by the Medical Research Council (MRC) and the UK Department for International Development (DFID) under the MRC/DFID Concordat agreement and is also part of the The European & Developing Countries Clinical Trials Partnership (EDCTP2) programme supported by the European Union. The authors acknowledge the support of the NIHR Oxford Biomedical Research Centre and the NIHR Thames Valley and South Midlands Clinical Research Network. V. E. P. is supported by the Bill & Melinda Gates Foundation (OPP1116967, OPP1151153) and the Wellcome Trust (106158/Z/14/Z). A. J. P. is an NIHR senior investigator.

***Potential conflicts of interest.*** A. J. P. has previously conducted studies on behalf of Oxford University funded by vaccine manufacturers, but currently does not undertake industry-funded clinical trials. A. J. P. chairs the UK Department of Health’s Joint Committee on Vaccination and Immunisation and the EMA scientific advisory group on vaccines, is a member of the World Health Organization’s (WHO) Strategic Advisory Group of Experts. He has received grants from the Wellcome Trust, Bill & Melinda Gates Foundation, Medical Research Council, and NIHR Oxford Biomedical Research Centre during the conduct of the study and grants from Okairos and Pfizer outside the submitted work. All other authors: No reported conflicts. All authors have submitted the ICMJE Form for Disclosure of Potential Conflicts of Interest. Conflicts that the editors consider relevant to the content of the manuscript have been disclosed.
