# Supplementary material for: The Impact of Vaccination and Prior Exposure on Stool Shedding of Salmonella Typhi and Salmonella Paratyphi in 6 Controlled Human Infection Studies
Source: Clin Infect Dis. 2018 Sep 25;68(8):1265–73. doi: 10.1093/cid/ciy670 (PMC6452003; doi:10.1093/cid/ciy670)
Supplement: Supplementary Material [file ciy670_suppl_supplementary_material.docx]

1. Supplementary material

**S1 Text: Methods**

Healthy volunteers aged 18-60 were screened for eligibility to exclude participants with significant medical or psychiatric history; disorders of the biliary tree; residence in typhoid/paratyphoid endemic countries >6 months, and history of previous typhoid vaccination. The challenge agents were prepared on the morning of challenge from frozen GMP stocks administered within 2 hours of preparation. Participants drank 120ml sodium bicarbonate solution followed by the challenge agent, suspended in 30ml sodium bicarbonate buffer. After challenge, participants attended the outpatient clinic for a minimum of 14 days, where daily blood and stool cultures were collected. Participants were diagnosed with enteric fever on meeting a composite diagnostic endpoint comprising fever 38^o^C for ≥12hrs and/or S. (Para)Typhi bacteraemia detected ≥72hrs from challenge. Antibiotics (ciprofloxacin 500mg PO BD or azithromycin 500mg PO OD for 14 days) were initiated at the time of typhoid diagnosis or at day 14 for those not diagnosed. All participants were effectively treated for their acute infections and no chronic carriers were identified.

Typhoid challenge was performed using the Qualies strain (genotype 3.1.0), isolated from the gallbladder of a typhoid carrier in 1958. Paratyphoid challenge was performed using the S. Paratyphi A NVGH308 strain, isolated from a bacteraemic patient from Nepal in 2007. The challenge doses administered differed between studies.

All studies were approved by Oxfordshire Research Ethics Committee A (10/H0604/53; 11/SC/0302; 14/SC/0004; 14/SC/1204; 14/SC/1427; 16/SC/0358) and performed according to the provisions of the Declaration of Helsinki and Good Clinical Practice guidelines.

**Stool Culture**

Selenite broth was inoculated with ~1g faeces and mixed by vortex. An XLD Agar plate was directly inoculated with 10μL of the suspension, and after 18-24 hours incubation at 37°C the selenite was inoculated onto chromogenic agar (BBL CHROMagar Salmonella agar, BD Diagnostics) for the detection of Salmonella spp.

**S2 Text: SAS code**

**proc** **glimmix** data = all method=quad(qpoints=**30**) noclprint;

class uniqueid day vaccine study /ref=first;

model stoolpositive=day vaccine study / dist=b link=logit solution cl;

random intercept/subject=uniqueid;

output out=out1 pred(ILINK)=predmean; *output probabilities for plotting (inverse logit of the linear predictor);

lsmeans vaccine/pdiff cl;

**run;**

*plot probabilities;

**proc** **sgplot** data = out1;

loess y=predmean x=day/ group=vaccine;

**run**;

**Figure S1 Isolation of *S*. Typhi from stool cultures of controls and vaccinees following challenge with 10^5^ organisms of *S*. Typhi in controlled human infection studies in Maryland (1959-1975)**

**
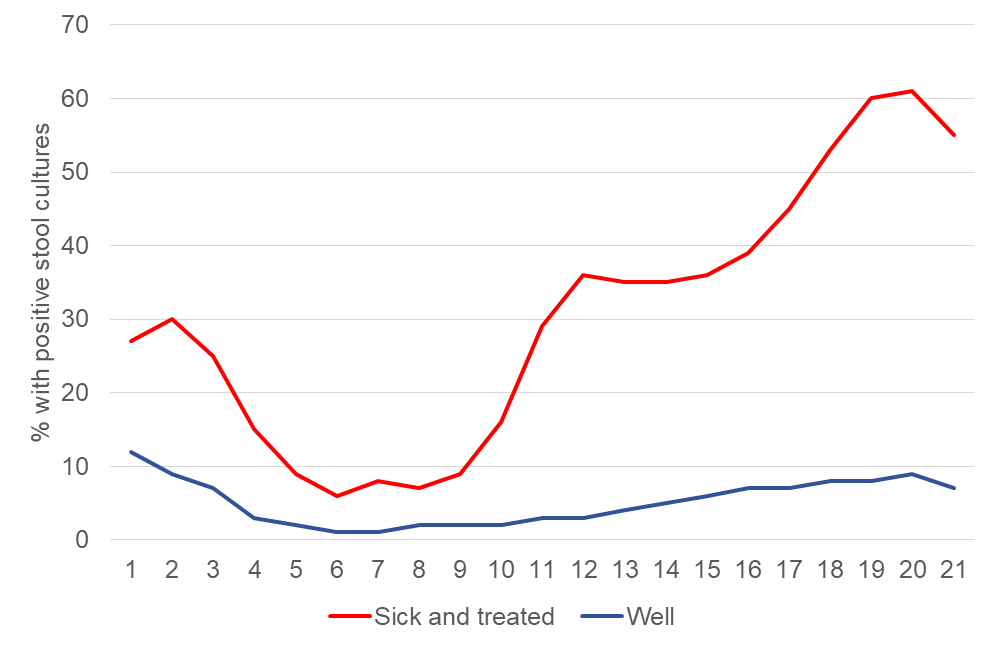
**

Reproduced with persmission from: “*Induced Typhoid Fever and Experimental Typhoid Vaccines: A study of 1886 volunteers.*” William E Woodward. University of Maryland. Unpublished Report.

**Sick and treated**: N=199 men. Data were included up to first day of treatment. Sick individuals were treated with antibiotics if their oral temperature reached 39.4°C (103°F) for two consecutive days. Antibiotics were given in an interrupted fashion, for 7 days, followed by 7 days of no treatment, followed by 5 days of treatment.

**Well**: participant’s temperature remained below 37.8°C (100°F)

Participants were adult male inmates of the House of Correction, Jessup, Maryland, who volunteered to participate in typhoid challenge studies conducted between 1959 and 1975.

**Findings**

“Stool cultures were performed routinely for 30 days on all recipients of 10^5^ challenge inoculum. There was a marked difference in results between volunteers who eventually developed illness requiring treatment and those who remained well. While remaining entirely well, up to 9% of volunteers had demonstrable levels of bacilli in their stool 3 weeks after challenge.

Of those developing treatable illness, there was no difference in the positivity of stool cultures in response to treatment with either chloramphenicol or ampicillin. There was a rebound in the number of positive cultures after cessation of therapy; this occurred twice when the treatment was interrupted.

It was not possible to determine directly the duration of the carrier state during the convalescence of all individuals since those with persistently negative cultures were no longer required to submit stool specimens after 30 days had elapsed. Only one patient became a long-term carrier. Following treatment, he remained well but with intermittently positive stool cultures for 746 days. His gallbladder was found to contain stones which had never caused symptoms and his bile harboured typhoid bacilli.

Prior military experience or receipt of an experimental vaccine was not associated with a decrease in isolation rate of typhoid bacilli from the faeces of men developing treatable illness”

William E Woodward, University of Maryland, 1975.

**Figure S2 Relationship between days of bacterial shedding in stool after S. Typhi challenge and antibody levels prior to challenge**

(A) Anti-*S*. Typhi LPS IgM prior to challenge with *S*.Typhi; (B) Anti-*S*. Typhi LPS IgA prior to challenge with *S*.Typhi; (C) Anti-Hd IgA prior to challenge with *S*. Typhi; (D) Anti-Vi IgM prior to challenge with *S*.Typhi. Days: y-axis represents the predicted total number of days of stool shedding (out of 14). The total number of days was determined from the logistic regression model by summing across all 14 days the predicted probability for each day for each person.


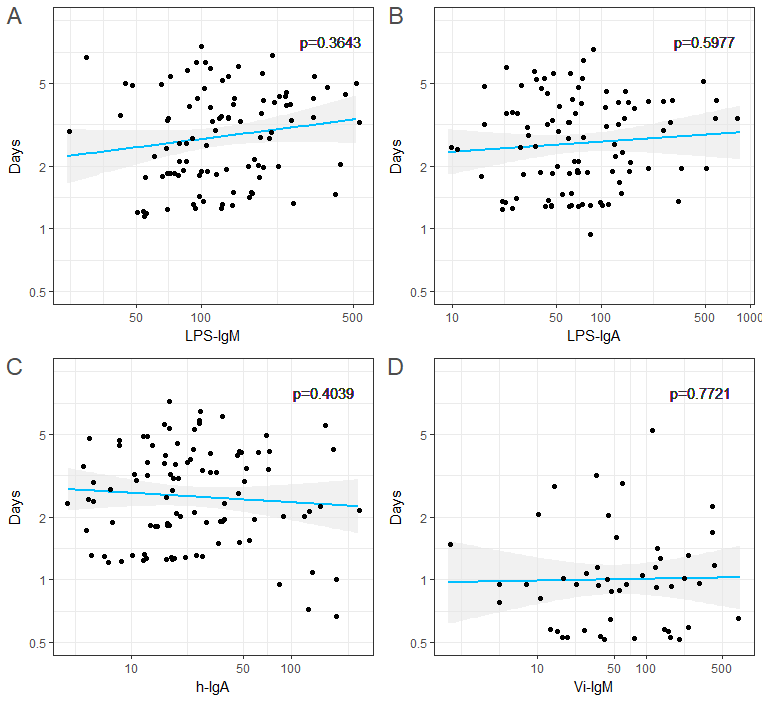


**Table S1 Bacterial shedding in stool after *S*. Typhi or *S*. Paratyphi challenge, according to previous exposure.**

| **Comparison** | **Odds Ratio** | **95% LCL** | **95% UCL** | **p value** |
| --- | --- | --- | --- | --- |
| P-P v T-P | 1.66 | 0.41 | 6.80 | 0.478 |
| P-P v P | 0.72 | 0.20 | 2.62 | 0.617 |
| P-T v T | 2.49 | 0.78 | 8.01 | 0.125 |
| P-T v T-T | 7.52 | 1.99 | 28.4 | 0.003 |
| T-T v T | 0.33 | 0.81 | 0.14 | 0.016 |
| T-P v P | 0.43 | 0.15 | 1.24 | 0.120 |

P = S. Paratyphi naïve (n=39); P-P = S.Paratyphi re-challenge after previous S. Paratyphi exposure (n=13); P-T = S.Typhi challenge after previous S.Paratyphi exposure (n=10); T = S. Typhi challenge in S. Typhi naïve participants (n=71); T-P = S. Paratyphi challenge after previous S. Typhi exposure (n=27); T-T = S. Typhi re-challenge after previous S. Typhi exposure (n=27).
